# 

**Published:** 1888-01-21

**Authors:** 


[illegible]o. 69, Vol. III—January 21st, 1888.

[Registered at the General Post Office as a Newspaper.]

Per Annum, 6s. 6d., post free.

Monthly Parts, 6d.; post free, 7½d.

Ditto per Ann., 7s. 6d. post free.

### THE HOSPITAL

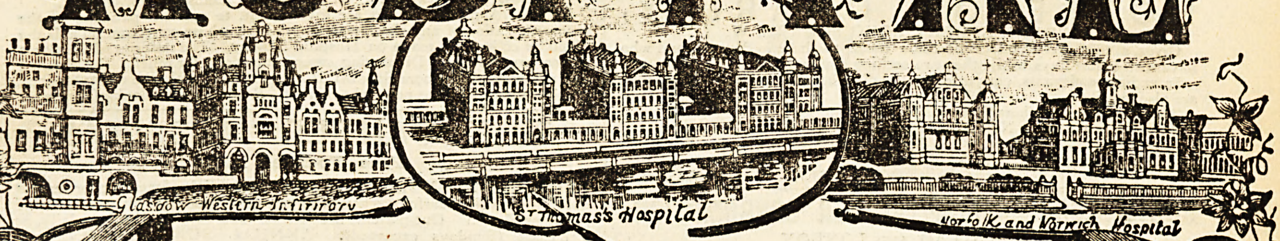

A WEEKLY INSTITUTIONAL JOURNAL OF

### Science, Medicine, and Philanthropy.

